# Etrasimod: Modulating Sphingosine-1-Phosphate Receptors to Treat Ulcerative Colitis

**DOI:** 10.3390/jcm14113890

**Published:** 2025-06-01

**Authors:** Cristina Martinez-Molina, Begoña González-Suárez

**Affiliations:** 1 Department of Pharmacy, Division of Medicines, Hospital Clínic de Barcelona, Villarroel 170, 08036 Barcelona, Spain; 2 Endoscopy Unit, Department of Gastroenterology, Clinical Institute of Digestive and Metabolic Diseases (ICMDM), Hospital Clínic de Barcelona, Villarroel 170, 08036 Barcelona, Spain

**Keywords:** etrasimod, sphingosine-1-phosphate receptor, ulcerative colitis, inflammatory bowel disease, immune-mediated disease, gastroenterology

## Abstract

This review aimed to provide a comprehensive overview of the current landscape of etrasimod. Etrasimod is an oral, once-daily selective modulator of sphingosine 1-phosphate receptors (S1PR), developed for the treatment of moderately to severely active ulcerative colitis and currently being explored for its potential in other immune-mediated inflammatory diseases. It selectively targets the S1PR subtypes S1PR_1_, S1PR_4_, and S1PR_5_, with limited activity on S1PR_3_ and no activity on S1PR_2_. Clinical trials have demonstrated that etrasimod significantly reduces symptoms and induces endoscopic improvement in patients with moderate to severe ulcerative colitis who are refractory or intolerant to at least one conventional therapy, biologic agent, or Janus kinase inhibitor, while maintaining a favourable safety profile. At the end of 2023, etrasimod was approved in the United States and Europe, and it is currently under review for ulcerative colitis in several other regions. Etrasimod offers a novel therapeutic option with unique characteristics that may help address the persistent unmet needs of real-world patients with moderately to severely active ulcerative colitis.

## 1. Introduction

Ulcerative colitis (UC), first described in 1859, is a chronic inflammatory bowel disease characterised by diffuse mucosal inflammation of the colon and rectum, with a relapsing-remitting course [1,2,3,4,5]. The most common symptom of UC is bloody diarrhoea, although the diagnosis is based on a combination of symptoms, endoscopic findings, and histological analysis. Its onset and progression follow a multifactorial pathogenesis involving genetic background, environmental and luminal factors, and mucosal immune dysregulation [1,2,3,4,5]. UC was estimated to affect 5 million people worldwide in 2023, with its incidence continuing to increase across the world [2].

The primary goal of UC treatment is to induce clinical remission and normalise biomarkers, followed by maintaining remission and enhancing the endoscopic appearance of the mucosa [2,6]. Current clinical practice guidelines recommend aminosalicylates as the first-line treatment for mild to moderate UC, with corticosteroids indicated for patients who do not respond adequately [7]. In moderate to severe UC, the therapeutic options have expanded with the approval of biologic agents and small-molecule drugs. These options include tumour necrosis factor (TNF) inhibitors (infliximab [8], adalimumab [9], golimumab [10]), an integrin inhibitor (vedolizumab [11]), an interleukin (IL)-12/23 inhibitor (ustekinumab [12]) and IL-23 inhibitors (mirikizumab [13], risankizumab [14]), Janus kinase (JAK) inhibitors (tofacitinib [15], upadacitinib [16], filgotinib [17]), and sphingosine-1-phosphate receptor (S1PR) modulators (ozanimod [18], etrasimod [19]).

Since the early 2000s, the introduction of biologic drugs and, more recently, JAK inhibitors in the management of UC have significantly improved treatment outcomes. However, there are still patients who either fail to respond (primary failure), experience adverse events, or show a decline in response over time (secondary failure) with these treatments. This underscores the need for effective and safe alternative therapies for the management of the disease.

Etrasimod is an oral, once-daily small-molecule drug (<1 kDa) that acts as a selective modulator of S1PR [19,20]. It selectively targets the receptor subtypes S1PR_1_, S1PR_4_, and S1PR_5_, with limited activity on S1PR_3_ and no activity on S1PR_2_ [19,20]. Clinical trials have demonstrated that etrasimod significantly reduces symptoms and induces endoscopic improvement in patients with moderate to severe UC who are refractory or intolerant to at least one conventional therapy, biologic agent, or JAK inhibitor, while maintaining a favourable safety profile [21]. Etrasimod was approved by the U.S. Food and Drug Administration (FDA) in October 2023 [20] and by the European Medicines Agency (EMA) in December 2023 [19], becoming one of the most recently available small-molecule drugs for the treatment of UC in clinical practice.

The aim of this review is to provide a comprehensive overview of etrasimod in the treatment of UC, highlighting its potential as a novel therapeutic option with distinct characteristics that may help address the persistent unmet needs of real-world patients with moderately to severely active UC.

## 2. Modulating Sphigosine-1-Phosphate Receptors

It has been suggested that UC patients exhibit increased levels of inflammatory T cells in the gastrointestinal tract [22]. In the early stages of UC, pro-inflammatory effector cells migrate to lymphoid tissues and present antigens to lymphocytes. The differentiated lymphocytes migrate from the lymph nodes to the intestinal mucosa, where they drive the proliferation and activation of inflammatory cells.

Sphingosine-1-phosphate is an extracellular signalling molecule derived from cell membrane sphingolipids [22,23]. It is involved in the migration of lymphocytes, including T cells, from the lymph nodes to tissues. After naive T cells are activated in the lymph nodes, they migrate toward an increasing S1P gradient [24]. S1P can interact with five distinct G-protein-coupled receptor subtypes (S1PR_1–5_), each with a determined tissue distribution pattern [25]. S1PR_1_ is ubiquitously expressed, including on the surface of lymphocytes [25,26,27]. Elevated expression of S1PR_1_ in colonic biopsies from patients with UC has been associated with increased intestinal vascularization at affected sites, a characteristic feature of UC-related inflammation [28].

Modulating S1PR reversibly retains specific lymphocytes in the lymph nodes and other secondary lymphoid tissues, thereby reducing their migration to regions of inflammation [22,29,30,31]. Fingolimod, a first-generation non-selective S1PR modulator drug, was approved by the FDA in 2010 for the treatment of relapsing-remitting multiple sclerosis [32]. The promiscuous binding of fingolimod to various S1PR subtypes, including S1PR_2_ and S1PR_3_, has been linked to serious adverse events, such as impaired pulmonary function, macular oedema, cardiovascular disorders, and malignancies [32,33,34]. Since then, a range of S1PR modulators has been developed and are being designed to treat other immune-mediated inflammatory diseases [22,35,36]. This includes prodrug agents including fingolimod, second-generation modulator drugs that closely mirror the canonical S1P molecule, and selective modulator drugs with enhanced safety profiles.

S1PR modulators have emerged as a hopeful therapeutic strategy in inflammatory bowel disease, including the approval of ozanimod and etrasimod (Table 1) for moderate to severe UC treatment [18,19,20,37].

Ozanimod, a selective S1PR_1,5_ modulator drug, is also approved for relapsing-remitting multiple sclerosis [18,37]. By targeting only S1PR_1_ and S1PR_5_, it avoids the complications associated with S1PR_2_ and S1PR_3_ modulation [38]. However, initiation of treatment with ozanimod requires an initial up-titration dosing regimen to gradually increase the dose (Table 1) and reduce the risk of a decrease in heart rate [18,37].

## 3. The Small-Molecule Drug Etrasimod

**Figure 1:**
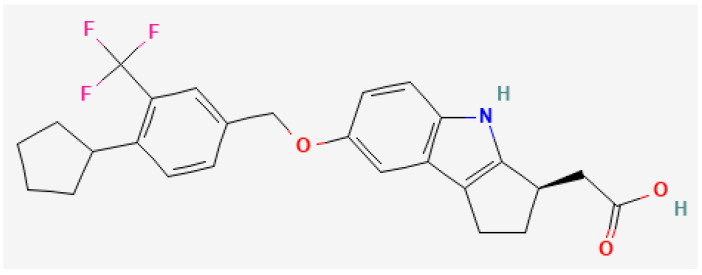
Chemical structure of etrasimod [40].

Etrasimod (Figure 1) is an oral, once-daily, selective S1PR_1,4,5_ modulator drug [19,20]. It acts as an agonist of S1PR_1_ and as a partial agonist of S1PR_4,5_, with no detectable effects on S1PR_2,3_ [39].

**Figure 2:**
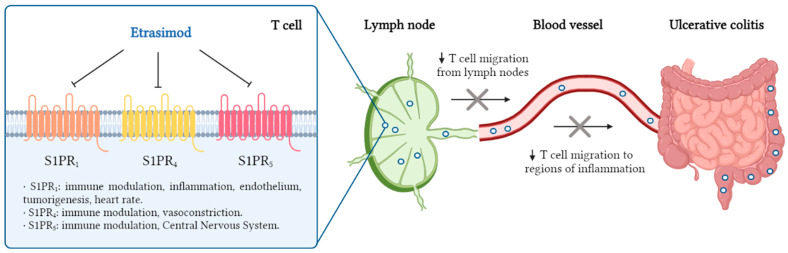
Mechanism of action of etrasimod. Etrasimod selectively modulates S1PR_1,4,5_, preventing T cell migration from lymph nodes to regions of inflammation in UC. Created in https://www.biorender.com (accessed on 19 January 2025).

Etrasimod reversibly inhibits lymphocyte egress from lymph nodes, leading to a dose-dependent reduction in T cell migration to regions of inflammation (Figure 2).

### 3.1. Pharmacodinamic Effects

Etrasimod reduces peripheral lymphocytes, with a greater impact on adaptive immune cells linked to UC, while having minimal impact on innate immune cells involved in immunosurveillance [20]. Etrasimod has been demonstrated to reduce lymphocyte levels to around half of their baseline value at 2 weeks of treatment, with effects maintained throughout the duration of therapy [21]. It results in a reduction in peripheral blood B cells (CD19+), T cells (CD3+), as well as T-helper (CD3+CD4+) and cytotoxic T lymphocyte populations (CD3+CD8+), whereas natural killer cells and monocytes remain unaltered. T-helper cells exhibit greater sensitivity to etrasimod compared to cytotoxic T lymphocytes. Following discontinuation, lymphocyte levels in peripheral blood return to baseline in a median of 2.6 weeks, with 90% of patients achieving normal levels within 4.7 weeks [20]. Etrasimod has been shown to reduce neutrophil counts, with levels generally remaining within the normal range. This reduction is reversible upon treatment discontinuation [20]. Due to its effect of reducing white blood cell levels, particularly lymphocytes, etrasimod treatment may increase susceptibility to infections [19,20]. Patients without a confirmed history of varicella or documented vaccination against varicella-zoster virus should be screened for antibodies prior to initiating etrasimod [20]. Vaccination is recommended for seronegative individuals, with etrasimod treatment deferred for 4 weeks post-vaccination to ensure adequate immune response [19,20].

Initiation of etrasimod therapy may cause transient reductions in heart rate and delays in atrioventricular conduction [19,20]. Assessment of pre-existing cardiac abnormalities via electrocardiogram is recommended for all patients [19,20]. Patients with specific pre-existing conditions are advised to undergo monitoring during the first dose of etrasimod [19,20]. Additionally, caution is advised when initiating etrasimod in patients treated with beta-blockers due to potential additive bradycardic effects [19,20]. Similar caution applies to patients receiving calcium channel blockers, QT-prolonging agents, and Class Ia or Class III antiarrhythmic drugs, as co-administration may enhance these effects [19,20].

The use of etrasimod during pregnancy may pose a risk to the developing foetus and is consequently contraindicated [19,20]. Women of child-bearing potential must use effective contraception during treatment and for at least 14 days after stopping etrasimod therapy [19].

### 3.2. Pharmacokinetic Properties

The currently approved dosage form of etrasimod is a 2 mg film-coated tablet [19,20]. Etrasimod can be administered with or without food, as food intake does not affect its pharmacokinetic parameters. However, administration with food is recommended during the first 3 days of treatment to mitigate potential transient reductions in heart rate associated with treatment initiation [19]. Treatment with etrasimod is initiated with a 2 mg once-daily dose, without the need for titration, and is maintained at the same dose throughout the maintenance of treatment. The time to achieve maximum plasma concentrations after oral administration is approximately 4 h (range: 2–8 h) [19]. Plasma concentrations at steady state are achieved within 7 days of administering 2 mg once daily [19].

Etrasimod undergoes extensive metabolism via cytochrome P450 (CYP) enzymes, predominantly CYP2C8 (38%), CYP2C9 (37%), and CYP3A4 (22%), with limited involvement of CYP2C19 and CYP2J2 [19]. Unmetabolized etrasimod constitutes the primary component detected in plasma, along with its primary metabolites M3 and M6. Etrasimod is responsible for over 90% of the activity at the S1P receptor [19]. Metabolism involves oxidation, dehydrogenation, and conjugation via uridine diphosphate glucuronosyltransferases (UGTs) and sulfotransferases.

The mean elimination half-life is approximately 30 h [19]. Excretion occurs primarily through the hepato-biliary route, with 82% eliminated in faeces and 4.89% through renal clearance [19].

Etrasimod exhibits consistent pharmacokinetics without significant variation based on sex, age (16 to ≥65 years old), race, or ethnicity [19]. No dose adjustment is required in patients > 65 years, but cautious use is advised owing to limited data and a potentially higher risk of adverse events [19].

No dose modification is required for patients with renal impairment, as etrasimod exposure measures are similar in those with severe renal impairment and normal renal function [19].

Etrasimod is contraindicated in severe hepatic impairment, but no adjustments are necessary for mild or moderate hepatic impairment [19].

### 3.3. Clinical Efficacy and Safety

The efficacy and safety of etrasimod in UC patients are primarily supported by two independent randomised, multicentre, double-blind, placebo-controlled, phase III trials: ELEVATE UC 52 and ELEVATE UC 12 [21].

The trials enrolled patients aged 16–80 years who had been diagnosed with UC for at least 3 months before screening, confirmed by endoscopy and histopathology, with disease extending ≥10 cm from the anal margin. Patients with isolated proctitis (rectal involvement < 10 cm) were also included and accounted for up to 15% of total enrolment.

Eligible patients presented a modified Mayo score (mMS) ranging from 4 to 9, an endoscopic subscore (ES) ≥ 2, and a rectal bleeding subscore (RBS) ≥ 1. All participants had a history of inadequate response, loss of response, or intolerance to at least one approved treatment for UC: oral aminosalicylates, corticosteroids, thiopurines, JAK inhibitors, or a biologic (e.g., TNF inhibitors, IL-12/23 inhibitor, integrin inhibitor).

The mean age of the participants enrolled in both trials was 40 years, with 3 patients (0.4%) under 18 years old and 45 patients (6%) aged 65 or older. The cohort was 57% male, 82% White, and 13% Asian.

In both studies, patients were randomised 2:1 to receive 2 mg of oral etrasimod daily or placebo. Participants were permitted to received concomitant UC therapies: stably doses of oral aminosalicylates and/or oral corticosteroids (≤ 20 mg of prednisone, ≤ 9 mg of budesonide, or equivalent). However, concomitant use of immunomodulators, biologic therapies, rectal 5-aminosalicylic acid, or rectal corticosteroids was not allowed.

ELEVATE UC 52 included 289 participants randomised to etrasimod and 144 to placebo, with a 12-week induction phase followed by a 40-week maintenance phase using a treat-through design. Of these, 91.7% of etrasimod patients and 86.1% of placebo patients completed Week 12. From Week 12 onward, patients with no improvement or disease worsening could discontinue and enter the open-label extension. By Week 52, 55.7% of etrasimod patients and 31.9% of placebo patients had completed treatment.

ELEVATE UC 12 enrolled 238 participants to etrasimod and 116 to placebo, consisting of a 12-week induction phase. Of these, 89.5% of etrasimod patients and 88.8% of placebo patients completed Week 12.

The primary endpoints in ELEVATE UC 52 were the proportion of patients achieving clinical remission at Weeks 12 and 52, while in ELEVATE UC 12, the primary endpoint was clinical remission at Week 12. Secondary endpoints included endoscopic improvement, symptomatic remission, and endoscopic improvement with histologic remission. Safety outcomes were evaluated in both trials.

In ELEVATE UC 52 and ELEVATE UC 12, a higher proportion of participants treated with etrasimod achieved clinical remission, endoscopic improvement, symptomatic remission, and endoscopic improvement with histologic remission at the defined time points, compared to those receiving placebo (Table 2).

Adverse events occurred in 206 (71%) of 289 patients in the etrasimod group and 81 (56%) in the placebo group in ELEVATE UC 52, and in 112 (47%) of 238 patients in the etrasimod group and 54 (47%) in the placebo group in ELEVATE UC 12 (Table 3). Serious adverse events were rare and occurred at similar rates in both treatment groups across the studies. Most adverse events were mild or moderate in severity. The most common adverse events (reported in ≥ 1% of patients) included anaemia, headache, and worsening or flare-ups of UC. In both trials, the rates of overall infections, serious infections, and opportunistic infections (e.g., tuberculosis and cytomegalovirus) were comparable between the etrasimod and placebo groups. No deaths or malignancies were reported in either study. Bradycardia or sinus bradycardia was reported in nine etrasimod-treated patients across both trials, with no events in the placebo group. Eight events occurred on day 1, and one on day 2. Two symptomatic events led to study discontinuation but resolved without treatment. No events of Mobitz type II or higher and no heart rates below 40 beats per minute were reported.

Across ELEVATE UC 52 and ELEVATE UC 12, macular oedema was reported in two patients receiving etrasimod and one patient receiving placebo. One patient receiving etrasimod discontinued treatment because of the event, while the other continued without interruption. All cases of macular oedema were resolved.

### 3.4. Current Clinical Trials

Some clinical trials are currently underway to evaluate etrasimod for other indications [41]. These include Phase II/III trials for Crohn’s disease and atopic dermatitis, and Phase II trials for eosinophilic oesophagitis [42]. Some indications have reached advanced development phases but have been discontinued, including alopecia areata, primary biliary cirrhosis, and pyoderma gangrenosum [42].

## 4. Etrasimod in the Treatment of Ulcerative Colitis

Among the therapeutic options currently approved for moderate to severe UC, S1PR modulators and JAK inhibitors constitute the only targeted synthetic therapies available. Since 2022, the EMA has recommended restricting the use of JAK inhibitors in specific patient populations. These include individuals aged 65 years or older, those with an increased risk of major cardiovascular events or malignancy, and individuals who smoke or have a history of long-term smoking [43]. This recommendation followed a review of data, including findings from a clinical trial with tofacitinib [44].

Targeted synthetic therapies are administered orally, providing an advantage for patients, particularly when compared to biologic agents that require intravenous or subcutaneous administration. Intravenous administration requires frequent hospital visits and incurs associated costs, as well as the need for specialised infusion staff. In contrast, oral administration provides greater convenience and flexibility for the patient. The seven biologic drugs currently approved for UC treatment are antibodies. As exogenous proteins, in contrast to targeted synthetic therapies, biologic agents can trigger immunogenicity, particularly TNF inhibitors drugs [45]. This may result in a loss of response, requiring adjustments in posology or the use of concomitant immunosuppressors (e.g., thiopurines), which in turn increases the risk of adverse events.

S1PR modulator drugs, such as etrasimod, offer a novel therapeutic option with a distinctive set of characteristics, which may address unmet needs of real-world patients with moderately to severely active UC.

## 5. Conclusions

Etrasimod, a novel S1PR modulator small-molecule drug, has shown promising efficacy and safety in the treatment of immune-mediated inflammatory diseases, particularly in moderate to severe UC. In the ELEVATE UC 52 and the ELEVATE UC 12 trials, it demonstrated efficacy comparable to established targeted therapeutic classes for UC treatment, such as TNF inhibitors, integrin inhibitors, IL-12/23 inhibitors, and JAK inhibitors, while maintaining a favourable safety profile. By the end of 2023, both the FDA and the EMA approved etrasimod for the treatment of moderate to severe UC. Certain special warnings and precautions should be considered during etrasimod treatment, including risks of infections and cardiac conduction abnormalities such as bradycardia and atrioventricular conduction delays. Also, careful consideration of potential drug–drug interactions is essential when prescribing concomitant treatments to ensure optimal clinical outcomes. In addition to its therapeutic efficacy and safety, etrasimod offers several advantages over some existing drug therapies, including a lack of immunogenicity, once-daily oral administration, rapid onset of action, a short half-life allowing for a quick wash-out period, and adequacy for use in the elderly population as well as in patients with contraindication to biologics. Overall, etrasimod represents a promising therapeutic option with unique characteristics that may address unmet needs of patients with moderately to severely active UC. Long-term real-world data will be essential to further elucidate its effectiveness and safety profile.

## Figures and Tables

**Table 1:** Main characteristics of ozanimod and etrasimod [18,19,20,37].

	Therapeutic Indications	Mechanism of Action	Method of Administration	Posology	Safety Considerations
**Ozanimod**	Multiple sclerosis. Ulcerative colitis.	Selective S1PR_1,5_ modulator.	Oral use. Can be taken with or without food.	Days 1–4: 0.23 mg once daily. Days 5–7: 0.46 mg once daily. Days 8 and thereafter: 0.92 mg once daily.	Bradyarrythmia and atrioventricular conduction delays. Liver injury. Infections. Hypertension. Macular oedema. Respiratory effects. Malignancies. PRES.
**Etrasimod**	Ulcerative colitis.	Selective S1PR_1,4,5_ modulator.	Oral use. Can be taken with or without food (co-administration with food is recommended for the first 3 days).	2 mg once daily.	

S1PR: sphingosine-1-phosphate receptor; PRES: posterior reversible encephalopathy syndrome.

**Table 2:** Primary and secondary endpoints from the ELEVATE UC 52 and ELEVATE UC 12 trials [21].

		Primary Endpoint		Secondary Endpoints					
		Clinical Remission *n* (%)		Endoscopic Improvement *n* (%)		Symptomatic Remission *n* (%)		Endoscopic Improvement with Histologic Remission *n* (%)	
		Week 12	Week 52	Week 12	Week 52	Week 12	Week 52	Week 12	Week 52
**ELEVATE UC 52** **(*n* = 433)**	**Placebo** **(*n* = 135)**	10 (7%)	9 (7%)	19 (14%)	11 (8%)	29 (21%)	19 (13%)	6 (4%)	28 (19%)
	**Etrasimod 2 mg** **(*n* = 274)**	74 (27%)	88 (32%)	96 (35%)	94 (33%)	126 (46%)	113 (39%)	58 (21%)	127 (44%)
	***p*** **-Value**	*p* < 0.0001	*p* < 0.0001	*p* < 0.0001	*p* < 0.0001	*p* < 0.0001	*p* < 0.0001	*p* < 0.0001	*p* < 0.0001
**ELEVATE UC 12** **(*n* = 354)**	**Placebo** **(*n* = 112)**	17 (15%)		21 (19%)		33 (29%)		10 (9%)	
	**Etrasimod 2 mg** **(*n* = 222)**	55 (25%)		68 (31%)		104 (47%)		36 (16%)	
	***p*** **-Value**	*p* = 0.026		*p* = 0.0092		*p* = 0.0013		*p* = 0.036	

**Table 3:** Summary of adverse events in the ELEVATE UC 52 and ELEVATE UC 12 trials [21].

	ELEVATE UC 52		ELEVATE UC 12	
	Etrasimod Group (*n* = 289) *n* (%)	Placebo Group (*n* = 144) *n* (%)	Etrasimod Group (*n* = 238) n (%)	Placebo Group (*n* = 116) *n* (%)
**Any AE**	206 (71%)	81 (56%)	112 (47%)	54 (47%)
**Any serious AE**	20 (7%)	9 (6%)	6 (3%)	2 (2%)
**Any AE leading to treatment discontinuation**	12 (4%)	7 (5%)	13 (5%)	1 (1%)
**AE leading to death**	0	0	0	0
**Most common AE**				
**Worsening of UC or flare**	22 (8%)	13 (9%)	9 (4%)	1 (1%)
**Anaemia**	24 (8%)	14 (10%)	14 (6%)	8 (7%)
**Headache**	24 (8%)	7 (5%)	11 (5%)	2 (2%)
**Nausea**	9 (3%)	2 (1%)	10 (4%)	2 (2%)
**COVID-19**	20 (7%)	9 (6%)	3 (1%)	3 (3%)
**Dizziness**	15 (5%)	1 (1%)	3 (1%)	0
**Pyrexia**	14 (5%)	6 (4%)	8 (3%)	3 (3%)
**Arthralgia**	13 (4%)	3 (2%)	4 (2%)	3 (3%)
**Abdominal pain**	11 (4%)	5 (3%)	3 (1%)	3 (3%)
**AE of special interest**				
**Serious infections**	3 (1%)	5 (3%)	0	0
**Herpes zoster**	2 (1%)	0	0	2 (2%)
**Opportunistic infections**	0	1 (1%)	1 (<1%)	0
**Hypertension**	8 (3%)	1 (1%)	3 (1%)	1 (1%)
**Sinus bradycardia**	0	0	4 (2%)	0
**Bradycardia**	4 (1%)	0	1 (<1%)	0
**AV block, 1st degree**	1 (<1%)	0	1 (<1%)	0
**AV block, 2nd degree (Mobitz type I)**	1 (<1%)	0	0	0
**Macular oedema**	1 (<1%)	0	1 (<1%)	1 (1%)

AE: adverse event; UC: ulcerative colitis; COVID-19: Coronavirus disease 2019; AV: atrioventricular.

## Data Availability

No new data were created or analysed in this study. Data sharing is not applicable to this article.

## Abbreviations

The following abbreviations are used in this manuscript:

UC	Ulcerative colitis
TNF	Tumour necrosis factor
IL	Interleukin
JAK	Janus kinase
S1PR	Sphingosine-1-phosphate receptor
FDA	U.S. Food and Drug Administration
EMA	European Medicines Agency
PRES	Posterior reversible encephalopathy syndrome
CYP	Cytochrome P450
UGTs	Uridine diphosphate glucuronosyltransferase
mMS	Modified Mayo score
ES	Endoscopic subscore
RBS	Rectal bleeding subscore
AE	Adverse event
COVID-19	Coronavirus disease 2019
AV	Atrioventricular
